# The Lords and the Hospitals

**Published:** 1890-12-06

**Authors:** 


### The Lords and the Hospitals.

Mr. Burdett, in the course of his paper, read at St. Thomas's Hospital on the 20th ult., stated: "Although the House of Lords Committee have not expressed any opinion, I feel called upon to say that the method of procedure so far adopted by that body is not calculated to bring out the true facts. In the first place, it must be patent to everybody that, if the discharged servants and nurses of every metropolitan and provincial hospital and poor-law institution are to be permitted to attend before the Lords Committee and to air their grievances, the work of this Committee will not be ended in our generation. I venture to think, and to say, that the only effectual way of sifting charges of this kind to the bottom is to request the complainants to formulate their grievances in writing, these should then be reduced to order under the authority of the Lords Committee, and a copy of them sent to the authorities of the institutions immediately concerned, with a request that they will give their answer in writing also. All the documents on both sides should be verified by statutory declaration. With the two statements before them, the Lords' Committee could decide on the facts, if any, and what further steps should be taken, and how far, if at all, evidence should be admitted on the points at issue. In this way much valuable time would be saved, none but carefully-prepared statements would be forthcoming, and so the Committee would be spared the oceans of voluble nonsense which they will otherwise have to face. Besides, the credit of the Committee is at stake, and for this reason, if for no other, it is to be hoped that the initial mistake will not be repeated when the sittings are resumed."

